# Superior Antitumor Activity of Nanoparticle Albumin-Bound Paclitaxel in Experimental Gastric Cancer

**DOI:** 10.1371/journal.pone.0058037

**Published:** 2013-02-27

**Authors:** Changhua Zhang, Niranjan Awasthi, Margaret A. Schwarz, Stefan Hinz, Roderich E. Schwarz

**Affiliations:** 1 Division of Surgical Oncology, Department of Surgery, The University of Texas Southwestern Medical Center, Dallas, Texas, United States of America; 2 Hamon Center for Therapeutic Oncology Research, Simmons Comprehensive Cancer Center, The University of Texas Southwestern Medical Center, Dallas, Texas, United States of America; 3 Department of Gastrointestinopancreatic Surgery, The First Affiliated Hospital of Sun Yat-Sen University, Guangzhou, Guangdong, China; 4 Department of Pediatrics, The University of Texas Southwestern Medical Center, Dallas, Texas, United States of America; University of Liverpool, United Kingdom

## Abstract

Gastric cancer is the second common cause of cancer related death worldwide and lacks highly effective treatment for advanced disease. Nab-paclitaxel is a novel microtubule-inhibitory cytotoxic agent that has not been tested in gastric cancer as of yet. In this study, human gastric cancer cell lines AGS, NCI-N87 and SNU16 were studied. Nab-paclitaxel inhibited cell proliferation with an IC50 of 5 nM in SNU16, 23 nM in AGS and 49 nM in NCI-N87 cells after 72-hour treatment, which was lower than that of oxaliplatin (1.05 μM to 1.51 μM) and epirubicin (0.12 μM to 0.25 μM). Nab-paclitaxel treatment increased expression of the mitotic-spindle associated phospho-stathmin irrespective of the baseline total or phosphorylated stathmin level, and induced mitotic cell death as confirmed through increased expression of cleaved-PARP and caspase-3. After a two-week nab-paclitaxel, oxaliplatin or epirubicin treatment, the average in vivo local tumor growth inhibition rate was 77, 17.2 and 21.4 percent, respectively (p = 0.002). Effects of therapy on tumoral proliferative and apoptotic indices corresponded with tumor growth inhibition data, while expression of phospho-stathmin also increased in tissues. There was an increase in median animal survival after nab-paclitaxel treatment (93 days) compared to controls (31 days, p = 0.0007), oxaliplatin (40 days, p = 0.0007) or to docetaxel therapy (81 days, p = 0.0416). The strong antitumor activity of nab-paclitaxel in experimental gastric cancer supports such microtubule-inhibitory strategy for clinical application. Nab-paclitaxel benefits were observed independent from phosphorylated stathmin expression at baseline, putting into question the consideration of nab-paclitaxel use in gastric cancer based on this putative biomarker.

## Introduction

Gastric cancer (GC) is the fourth most prevalent cancer and the second most common cause of cancer-related deaths throughout the world [1]–[3]. Most patients have advanced or metastatic disease at the time of diagnosis [4], [5]. The combination of oxaliplatin and 5-fluorouracil, with and without epirubicin, has become the most widely used regimen in first-line chemotherapy for advanced gastric cancer [6], [7]. However, this combination treatment has the potential for considerable side effects and still carries limited efficacy, while patients' prognosis remains dismal with a median survival of around 10 months [8]–[11]. In an effort to improve survival and to decrease the toxicity, molecularly targeted therapy is being actively investigated for the treatment of gastric cancer [12].

Microtubules have long been considered a target of interest for some anticancer drugs because of their universal role in proliferating cells and their essential role in mitosis [13]–[15]. Paclitaxel and docetaxel are classical microtubule inhibitors that exert their activity by promoting tubulin polymerization and stabilization of microtubules resulting in G2-M phase arrest and mitotic cell death [15]. Mitotic cell death is a mode of cell death occurring specifically during mitotic stages induced by DNA damaging agents and spindle poisons/mitotic inhibitors [16], [17]; it is mainly caspase dependent, but under rare circumstances can also be caspase independent [18]. Paclitaxel has been tested for advanced and recurrent gastric cancers with a response rate of 43% in combination with 5-fluorouracil and folinic acid [9], [19]. Compared to the response rate of 38∼45% yielded by a combination of oxaliplatin with 5-fluorouracil and folinic acid, paclitaxel did not result in superior survival but caused more side effects, especially in elderly patients [9], [20]–[22]. Paclitaxel required emulsification with solvents to allow intravenous administration which has resulted in hypersensitivity reactions and potentially dramatic side effects in patients [23], [24]. Nanoparticle albumin-bound (nab) paclitaxel is a novel albumin-stabilized, cremophor-free and water-soluble nanoparticle formulation of paclitaxel. It is well tolerated with no hypersensitivity reactions after intravenous infusion [25]. Clinical and experimental studies demonstrated that compared with solvent-based paclitaxel, nab-paclitaxel had higher tumor retention, lower toxicity [23], [26] and more potent antitumor effects on breast cancer, non-small cell lung carcinoma (NSCLC), pancreatic cancer, melanoma, and head and neck cancer [27]–[31]. However, the potential role of nab-paclitaxel in gastric cancer cells is not tested as of yet.

Stathmin, a microtubule-destabilizing phosphoprotein, is an important regulator of microtubule polymerization and dynamics [32]–[34], and was found to be related to taxane resistance in some tumor types through altering drug binding and delaying G2-M phase transition [33], [35]. Stathmin inhibition had a synergistic antiangiogenic and antitumor activity with taxol [36]. Stathmin expression has been found to be present in a wide variety of human cancers including gastric cancer, and therefore may represent an attractive target for cancer therapy [34], [37], [38]. Jeon et al. found that stathmin might serve as a prognostic marker and a potential therapeutic target for gastric cancer [37]. Phosphorylation of stathmin reduces its microtubule destabilizing effects, a phenomenon that has also been ascribed to taxane activity [34].

This study evaluated the antitumor activities of nab-paclitaxel in human gastric cancer cells in vitro and in vivo. We compared the antitumor efficacy of nab-paclitaxel to other cytotoxic agents on local tumor growth and animal survival. We also measured the expression of total stathmin and phospho-stathmin in vitro and vivo to assess their role as predictive markers in the nab-paclitaxel antitumor response.

## Materials and Methods

### Cell culture and reagents

The human gastric cancer cell lines AGS, NCI-N87 and SNU16 were obtained from the American Type Culture Collection (ATCC, Rockville, MD) and cultured in RPMI 1640 medium (Sigma Chemical Co. St. Louis, MO) supplemented with 10% fetal bovine serum (FBS) at 37°C in a humidified 5% CO_2_ atmosphere. Nab-paclitaxel was purchased from Abraxis BioScience (Los Angeles, CA). Oxaliplatin was purchased from Sanofi Aventis (Bridgewater, NJ). Docetaxel, epirubicin and 5-fluorouracil were obtained from a local pharmacy. The cell proliferation reagent WST-1 was purchased from Roche Diagnostic Corporation (Indianapolis, IN).

### Cell viability assay

Cell viability was evaluated by the colorimetric WST-1 assay. The measurement is based on the ability of viable cells to cleave the sulfonated tetrazolium salt WST-1 (4-[3-(4-iodophenyl)-2-(4-nitrophenyl)-2H-5-tetrazolio]-1, 3-benzene disulfonate) by mitochondrial dehydrogenases [39]. Gastric cancer cells (5,000 cells per well) were plated in a 96-well plate in regular growth medium and were treated with nab-paclitaxel, 5-fluorouracil, oxaliplatin and epirubicin after 16 hours incubation. The range of concentrations used (1 nM to 10 μM) was comparable to their clinically achievable concentrations. After additional incubation of 72 hours, 10 μl WST-1 reagent was added in each well followed by incubation for 2 hours. The absorbance at 450 nm was measured using a microplate reader.

### Cell cycle analysis

Cell cycle analysis was performed by flow cytometry with fluorescent propidium iodide (PI) staining. Cells were cultured and treated with nab-paclitaxel, oxaliplatin, epirubicin and docetaxel for 8 to 24 hours. Cells were then collected and fixed in 75% ethanol–PBS overnight at −20°C. Cells were centrifuged for 5 min at 2000×g. The cell pellet was resuspended and incubated in 0.05 mg/ml PI, 0.1% Triton X-100, and 1 mg/ml RNAse A in PBS for 45 min at room temperature. The suspension was then analyzed on a Becton Dickinson FACScan. The ratio of cells in the sub-G1, G1, S, and G2-M phases of cell cycle was determined by their DNA content.

### Immunocytochemical analysis

Gastric cancer cells (1×10^5^ cells per chamber) were plated in a 4-chamber slide in regular growth medium. After 24-hour the cells were treated with nab-paclitaxel for 16 hours and then fixed in 4% paraformaldehyde. Cells were incubated with CAS blocking buffer followed by 1-hour incubation with phospho-stathmin antibody (1∶100) and 40 minutes incubation with Cy3 (1∶200 dilution) secondary antibody. Slides were mounted using mounting solution containing 4′,6-diamidino-2-phenylindole (DAPI) (Invitrogen, Carlsbad, CA). Fluorescence microscopy was used to detect fluorescent signals using the IX81 Olympus microscope equipped with a Hamamatsu Orca digital camera (Hamamatsu Corporation, Bridgewater, NJ).

### Western blot analysis

Sub-confluent monolayers of cells were treated with nab-paclitaxel, oxaliplatin and epirubicin. Cell lysates and tumor lysates were obtained as previously described [40]. Supernatants were recovered by centrifugation at 13000 rpm, protein concentrations were measured and equal amounts of total protein were separated by SDS-PAGE. Proteins were transferred to PVDF membranes (Bio-Rad, Hercules, CA) and the membranes were blocked for 1 hour in TBS-T. The membranes were incubated overnight at 4°C with the following antibodies: total stathmin, phospho-stathmin (ser38), cleaved poly (ADP-ribose) polymerase-1 (PARP-1), cleaved caspase-3 (all from Cell Signaling Technology, Beverly, MA), α-tubulin and β-actin (both from Sigma, St. Louis, MO). The membranes were then incubated with the corresponding HRP-conjugated secondary antibodies (Pierce Biotechnologies, Santa Cruz, CA) for one hour. Specific bands were detected using the enhanced chemoilluminescence reagent (ECL, Perkin Elmer Life Sciences, Boston, MA) on autoradiographic film.

### Subcutaneous tumor growth study

All animal experiments were carried out in accordance with the guidelines and approved protocols of the University of Texas Southwestern Medical Center (Dallas, TX) Institutional Animal Care and Use Committee (Permit Number 2012–0081). The animals were monitored daily throughout the experiment for any sign of distress. Female SCID mice (6 to 8 weeks) were used for comparative modeling of subcutaneous tumor growth. Gastric cancer cells (10×10^6^ NCI-N87 or 20×10^6^ SNU16 cells) were subcutaneously injected into each mouse. Mice were weighed twice a week. Fourteen days after tumor cell injection, all mice had measurable tumor (an average tumor size of 100 to 150 mm^3^). Mice were then randomly grouped (n = 6∼8 per group) and treated intraperitoneally with PBS (control), nab-paclitaxel (10 mg/kg in 100 μl PBS, 2 times a week), oxaliplatin (5 mg/kg in 100 μl PBS, 2 times a week), and epirubicin (1 mg/kg in 100 μl PBS, 2 times a week) for 14 days. The tumor size was measured twice weekly via caliper, and tumor volume (V) was calculated by using the formula V = ½ [L × (W)^2^], L =  length and W =  width. Relative tumor volume (RTV) was determined according to the formula RTV  =  V_n_/V_0_ where V_0_ and V_1_ represent tumor volume at the Day 0 and the succeeding measurement day, respectively. The tumor growth inhibition rate was calculated by using the formula (1-RTVt/RTVc) ×100% where t and c represent treatment and control group. After completion of treatment, all mice were euthanized with CO2, tumors were removed, weighed, dissected and processed for histological, immunohistochemical and Western blot analysis.

### Immunohistochemical analysis

Tumor tissue specimens were fixed in 4% paraformaldehyde and embedded in paraffin. Intratumoral proliferation was measured by Ki67 nuclear antigen staining as per manufacturer's protocol (Abcam, Cambridge, MA). Paraffin-embedded tissue sections were cut (5 μm), deparaffinized, rehydrated and antigen retrieved. The tissue sections were incubated with CAS blocking buffer followed by 1-hour incubation with 1∶200 dilution of primary Ki67 or phospho-stathmin antibody (1∶200) and 40 minutes incubation with Cy3 (1∶200 dilution) secondary antibody. Slides were mounted using mounting solution containing 4′,6-diamidino-2-phenylindole (DAPI) (Invitrogen). Intratumoral proliferative index and stathmin activity was evaluated by calculating the positive cells from five different high-power fields (HPF) in a blinded manner. Intratumoral apoptotic activity was evaluated by staining tissue sections with “Apoptag Apoptosis Detection Kit” according to the manufacturer's (Millipore) instructions. Fluorescence microscopy was used to detect fluorescent signals using the IX81 Olympus microscope equipped with a Hamamatsu Orca digital camera (Hamamatsu Corporation, Bridgewater, NJ) and a DSU spinning confocal unit using Slidebook software (Intelligent Imaging Innovations, Philadelphia, PA).

### Animal survival analysis

Animal survival studies were performed using 6- to 8-week-old female SCID mice [41]. The mice were intraperitoneally injected with SNU16 (40×10^6^) cells and body weight was measured twice a week. Three weeks after tumor cell injection mice were randomly grouped (n = 6 to 8 per group).

In the first survival study, mice were treated intraperitoneally with PBS (control), nab-paclitaxel (10 mg/kg in 100 μl PBS, 2 times a week), and oxaliplatin (5 mg/kg in 100 μl PBS, 2 times a week) for 2 weeks. In the second survival study, mice were treated intraperitoneally with PBS (control), nab-paclitaxel (10 mg/kg in 100 μl PBS, 2 times a week), docetaxel (3 mg/kg in 100 μl PBS, 2 times a week) or oxaliplatin (5 mg/kg in 100 μl PBS, 2 times a week) for 2 weeks. Animal suffering was reduced by euthanizing when turning moribund according to predefined criteria including rapid weight loss or gain (>15%), failure to eat or drink, lethargy, inability to remain upright and lack of strength. Animal survival was evaluated from the first day of treatment until death.

### Statistical analysis

Survival analysis was evaluated with GraphPad Prism 5 Software (GraphPad Software, San Diego, CA). In vitro cell proliferation data are expressed as mean ± standard deviation. Statistical analysis was performed by ANOVA for multiple group comparison and Student's t-test for the individual group comparison. Survival group comparison was performed via log-rank test within a Kaplan-Meier type analysis. Values of p<0.05 were considered to represent statistically significant group differences.

## Results

### Stathmin expression in gastric cancer cells

**Figure 1:**
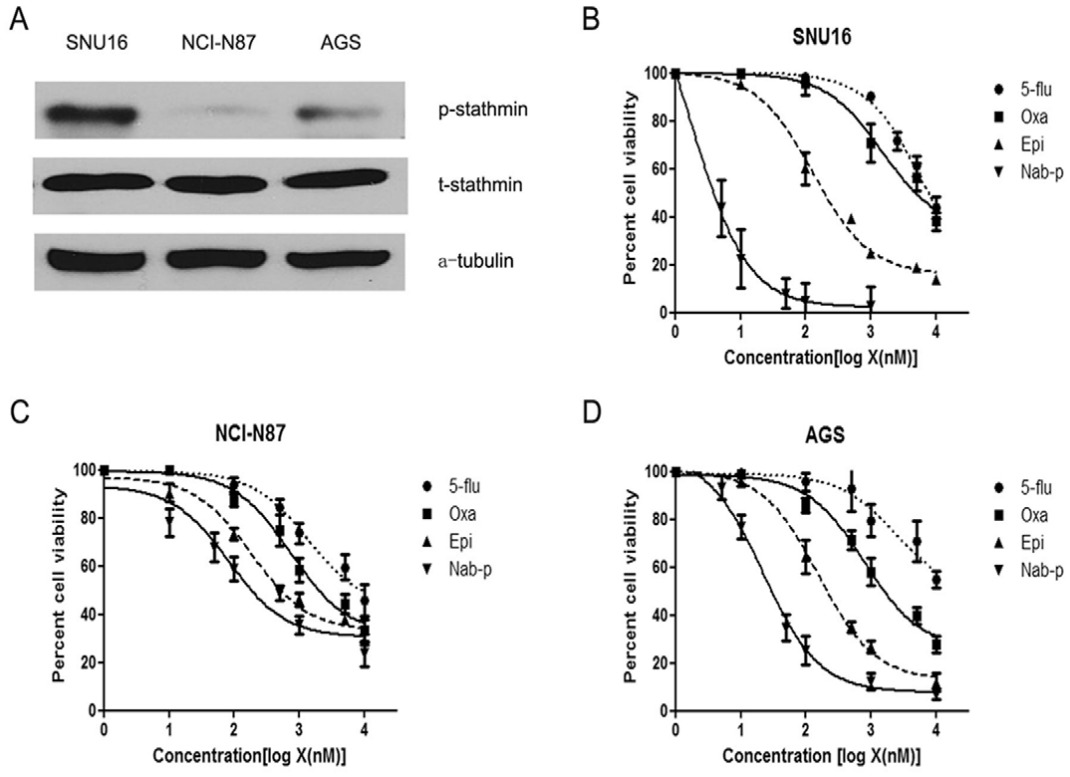
Nab-paclitaxel inhibits human gastric cancer cells proliferation. (A) Total cell extracts of gastric cancer cells were analyzed by immunoblotting for expression of stathmin and phospho-stathmin. (B–D) Human gastric cancer cells SNU16(B), NCI-N87 (C) and AGS (D) were plated on 96-well plates and treated with 1 nM to 1000 nM concentrations of nab-paclitaxel, 5-fluorouracil, oxaliplatin and epirubicin. After 72 hours, 10 μl WST-1 reagent was added in each well and incubated for 2 additional hours. The absorbance at 450 nm was measured using a microplate reader. The resulting number of viable cells was calculated by measuring absorbance of color produced in each well. Data are the mean ± SD of triplicate determinations.

Analysis of the human gastric cancer cells AGS, NCI-N87 and SNU16 revealed that all three lines expressed stathmin. No difference of total stathmin expression was found among these three cell lines. Expression of phospho-stathmin varied between cell lines, in the following order: SNU16> AGS > NCI-N87 cells (Figure 1A).

### Nab-paclitaxel inhibits gastric cancer cell proliferation

Nab-paclitaxel inhibited gastric cancer cell proliferation in a dose-dependent fashion, and inhibition in cell proliferation appeared to follow the same order as expression of phospho-stathmin (Figure 1B). At 100 nM concentration inhibition in cell proliferation was 94.5, 66.7 and 41.8 percent in SNU16, AGS and NCI-N87 cells respectively. Nab-paclitaxel showed the highest antiproliferative potency with a lowest effective dosage found in all 3 cell lines tested compared to 5-fluouracil, oxaliplatin and epirubicin. The IC_50_ of nab-paclitaxel was 5 nM in SNU16, 23 nM in AGS and 49 nM in NCI-N87 cells, which was less than 5-fluouracil (6.46 μM in SNU16, >10 μM in AGS and 10.2 μM in NCI-N87 cells), oxaliplatin (1.51 μM in SNU16, 1.64 μM in AGS and 1.05 μM in NCI-N87 cells) or epirubicin (0.12 μM in SNU16, 0.16 μM in AGS and 0.25 μM in NCI-N87 cells) (Figure 1C).

### Nab-paclitaxel induces G2 arrest and mitotic cell death in vitro

**Figure 2:**
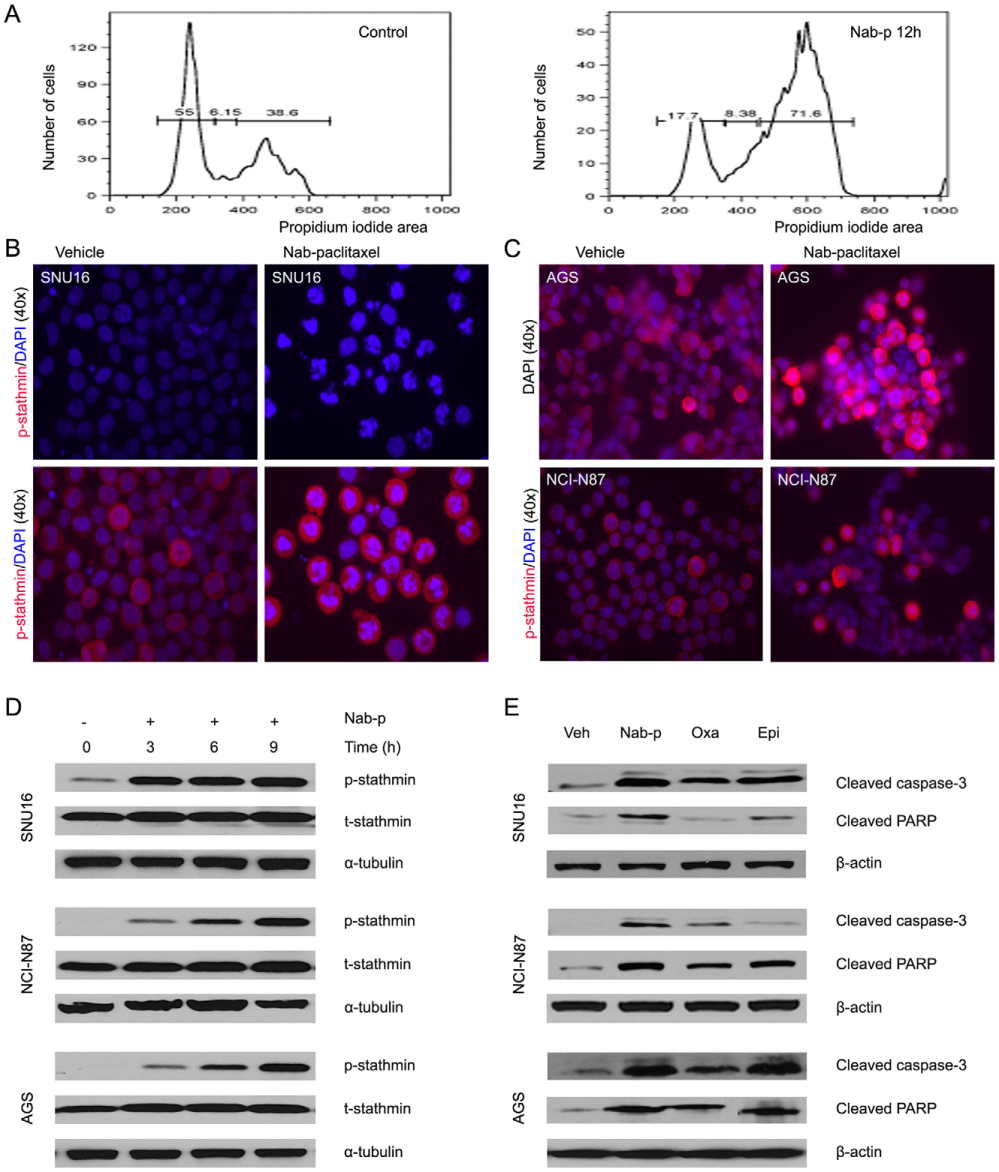
Effects of nab-paclitaxel on cell cycle progression, expression of phospho-stathmin and mitotic cell death in vitro. (A) SNU16 cells were cultured for 24 hours and then treated with 100 nM nab-paclitaxel for 12 hours. Cell cycle analysis was performed by flow cytometry. Results shown are representative of two independent experiments. (B–D) Expression of phospho-stathmin and mitotic cell death was evaluated in SNU16 (B), NCI -N87 (C), and AGS (C) cells. Cultured cells were treated with nab-paclitaxel (10 μM) and showed increased mitotic arrest configuration and phospho-stathmin expression by immunocytochemical staining. Mitotic arrests were detected in the presence of phospho-stathmin expression. (D) A sub-confluent monolayer of human gastric cancer cells AGS, NCI-N87 and SNU16 was treated with nab-paclitaxel (10 μM) for 3, 6 and 9 hours and analyzed by immunoblotting for phospho-stathmin and total stathmin. (E) Human gastric cancer cell were treated with nab-paclitaxel (10 μM), oxaliplatin (10 μM), and epirubicin (10 μM) for 16 hours and analyzed for cleaved PARP-1 and caspase-3. Data are representative of two independent experiments with similar results.

To evaluate the underlying mechanism of growth inhibition by nab-paclitaxel, the cell cycle profile was analyzed. As shown in Figure 2A, nab-paclitaxel (100 nM) resulted in G2-M cell cycle arrest in SNU16 cells. The percentage of propidium iodide positive cells in G2-M phase increased from 35.6 to 71.6 after 100 nM nab-paclitaxel treatment for 12 hours. Similar changes occurred in AGS and NCI-N87 cells with nab-paclitaxel and docetaxel treatment but not with oxaliplatin and epirubicin treatment (Figures S1 and S2).

Effect of nab-paclitaxel on mitotic cell death, expression of phospho-stathmin and apoptosis-related proteins was measured by immunocystochemistry and Western blot. Nab-paclitaxel treatment caused microtubule disruption and mitotic arrests in gastric cancer cells as observed by nucleus fragmentation and karyopyknosis (Figure 2B). Expression of phospho-stathmin was increased after nab-paclitaxel treatment, and higher expression of phospho-stathmin was associated with a greater degree of mitotic arrests, nucleus fragmentation or karyopyknosis, and cell death (Figure 2B and 2C). Nuclear fragmentation and karyopyknosis occurred in 16.7% of cells with low expression of phospho-stathmin but in 95.7% of cells with high expression of phospho-stathmin (r = 0.672, p<0.001). There was also evidence for time dependent increased expression of phospho-stathmin by nab-paclitaxel treatment (Figure 2D).

We examined if the mitotic cell death induced by nab-paclitaxel could in part be correlated with induction of apoptosis. Evaluation of PARP-1 cleavage and caspase-3 cleavage as markers of induction in apoptosis revealed that in gastric cancer cells nab-paclitaxel, oxaliplatin and epirubicin treatment caused an increase in the expression of both cleaved PARP-1 and caspase-3. This expression of apoptosis-related proteins appeared higher after nab-paclitaxel treatment compared with oxaliplatin or epirubicin treatments (Figure 2E).

### Nab-paclitaxel inhibits the growth of gastric cancer xenografts

**Figure 3:**
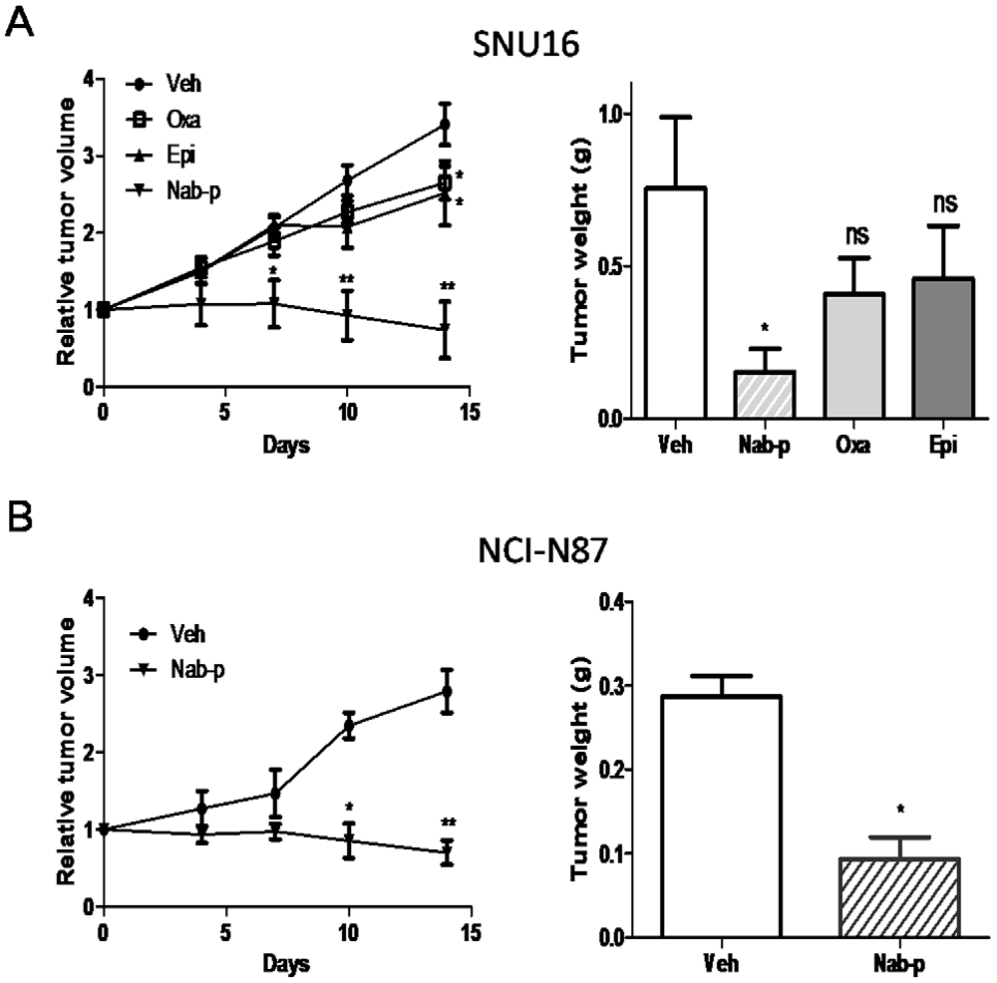
Nab-paclitaxel inhibits growth of established local tumor. (A) SCID mice were subcutaneously injected with SNU16 cells (20×10^6^) and treated with nab-paclitaxel, oxaliplatin and epirubicin for 2 weeks. (B) SCID mice were subcutaneously injected with NCI-N87 cells (10×10^6^) and treated with nab-paclitaxel for 2 weeks. Relative tumor volume and tumor weight on the final day were assessed. Data are representative of mean values ± standard deviation from 6–8 mice per group. Symbol * represents significant difference (p<0.05) and symbol ** represents significant differences (p<0.001) compared to vehicle controls; ns  =  no significant difference.

In vivo antitumor effects of nab-paclitaxel were evaluated in a murine xenograft model using SNU16 cells. Nab-paclitaxel therapy at 10 mg/kg twice a week for two weeks was well tolerated without obvious signs of toxicity as judged by mouse weight and daily assessment. After a two-week treatment, nab-paclitaxel therapy resulted in statistically significant tumor growth inhibition (p = 0.0004) which was greater than those of oxaliplatin (p = 0.0361) and epirubicin (p = 0.032) compared to vehicle control, respectively (Figure 3A). The tumor growth inhibition rate after a 2-week treatment with nab-paclitaxel, oxaliplatin and epirubicin was 77, 17.2 and 21.4 percent (p = 0.002), respectively. Only nab-paclitaxel treatment created a size reduction of subcutaneous lesions compared to their preestablished tumor size. Mean tumor weight was significantly decreased by nab-paclitaxel treatment (0.154±0.075 g vs. 0.756±0.232 g, p = 0.037), but not by oxaliplatin (0.408±0.12 g) or epirubicin (0.46±0.172 g) treatment compared to vehicle control, respectively (Figure 3A). We also evaluated the antitumor effects of nab-paclitaxel on NCI-N87 xenograft mouse local tumors. After two weeks of treatment, nab-paclitaxel therapy resulted in significant tumor volume inhibition (p = 0.0023) (Figure 3B) and the tumor growth inhibition rate was 72.8 percent. Mean tumor weight in the nab-paclitaxel treatment group was significantly lower than that in the vehicle group (0.093±0.026 g vs. 0.288±0.02 g, p = 0.0054) (Figure 3B). No significant change in mouse body weight was observed in any of the nab-paclitaxel, oxaliplatin or epirubicin therapy groups.

### Nab-paclitaxel induces mitotic cell death in vivo

**Figure 4:**
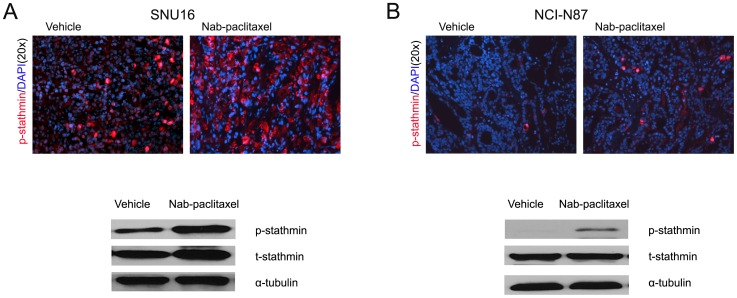
Effects of nab-paclitaxel treatment on expression of phospho-stathmin in vivo. SCID mice were subcutaneously injected SNU16 cells (20×10^6^) or NCI-N87 cells (10×10^6^) and treated with nab-paclitaxel for 2 weeks. Intratumoral expression of phospho-stathmin was measured in SNU16 (A) or NCI-N87 (B) tumor tissue by immunostaining tissue sections for phospho-stathmin (ser38) antigen and photographed under a fluorescent microscope. Tumor lysates were prepared from tumor tissue samples obtained from SNU16 (A) or NCI-N87 (B) tumor bearing SCID mice after nab-paclitaxel therapy and then analyzed by immunoblotting for phospho-stathmin and total stathmin.

Mechanisms of in vivo antitumor activity of nab-paclitaxel were further examined in tumor tissues obtained from SNU16 and NCI-N87 xenografts. A significant increase in the expression of phospho-stathmin was observed in SNU16 (Figure 4A) and NCI-N87 (Figure 4B) xenograft tumors with the treatment of nab-paclitaxel by both immunohistological and Western blot analyses.

**Figure 5:**
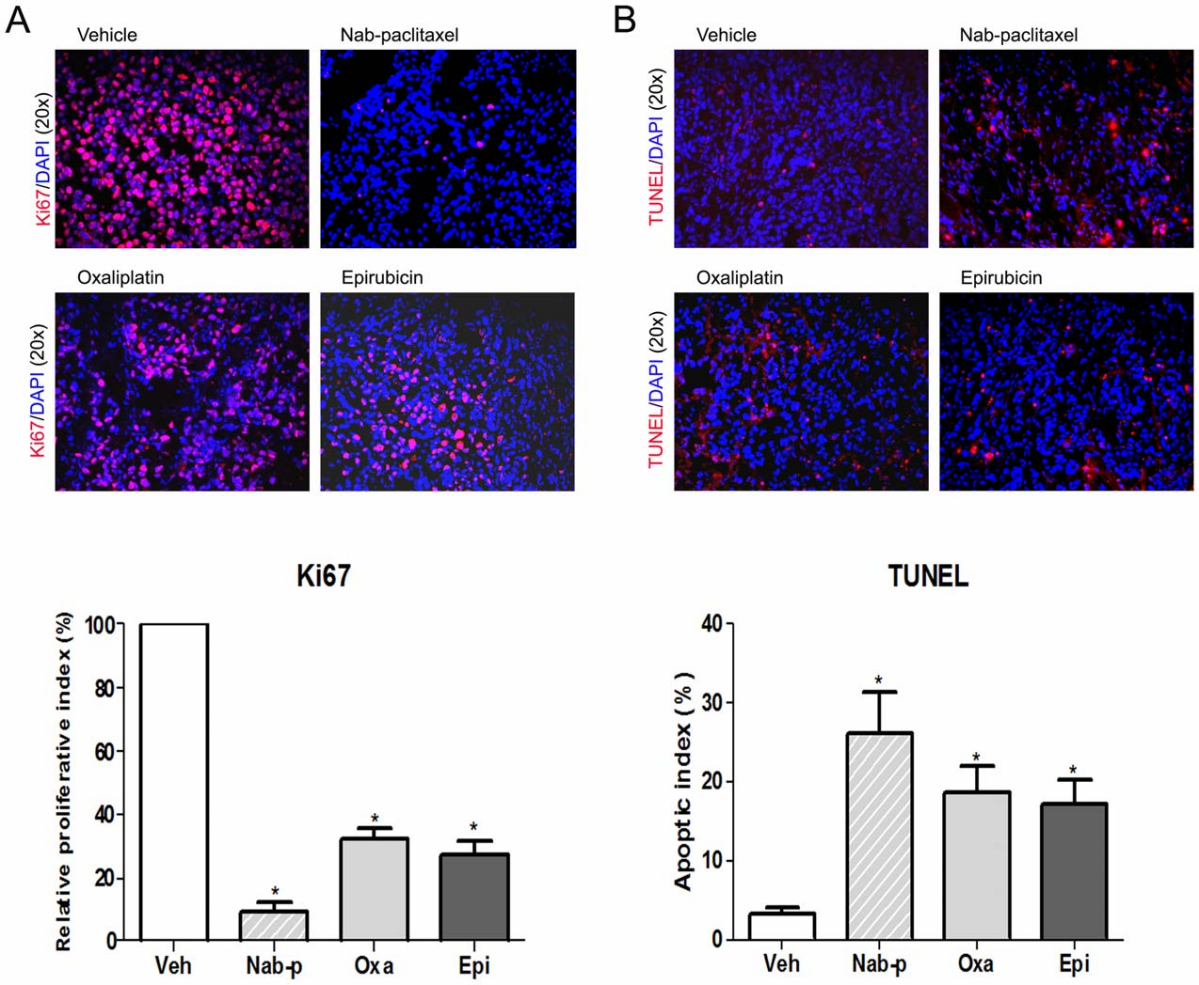
Effects of nab-paclitaxel treatment on cell proliferative and apoptotic activity in vivo. SCID mice were subcutaneously injected SNU16 cells (20×10^6^) and treated with nab-paclitaxel for 2 weeks. (A) Intratumoral proliferation was measured by immunostaining tissue sections with Ki67 nuclear antigen followed by fluorescent microscope photography. Ki67-positive cells were counted in five high power fields per sample. Fold change in proliferative index was standardized to controls (set to 1) and other samples are compared relative to this sample. (B) Intratumoral apoptosis was measured by staining tumor tissue section with the TUNEL procedure and subsequent fluorescent microscope photography. TUNEL-positive apoptotic cells were counted in five high power fields per sample. For both immunostaining experiments, each group had at least three samples counted and the data are expressed as the mean ± standard deviation. Symbol * represents significant difference compared to vehicle group (p<0.05).

Tumor tissue from nab-paclitaxel treated mice displayed a decreased tumor cell proliferation rate. A ki67 expression-based intratumoral proliferative index decreased by 90.7% (p = 0.001) compared to controls in the nab-paclitaxel treated group, as opposed to 72.6% (p = 0.004) in the oxaliplatin treated group and 67.7% (p = 0.003) in the epirubicin treated group (Figure 5A). Nab-paclitaxel caused a stronger inhibition effect on tumor cell proliferation than oxaliplatin and epirubicin.

Examination of apoptosis in tumor tissues revealed that the induction in apoptotic index was 7.9-fold in the nab-paclitaxel group (p = 0.013), 5.7-fold in oxaliplatin treated animals (p = 0.024), and 5.2-fold in the epirubicin treated group (p = 0.042) over the baseline within the control group (Figure 5B).

### Nab-paclitaxel increases animal survival

**Figure 6:**
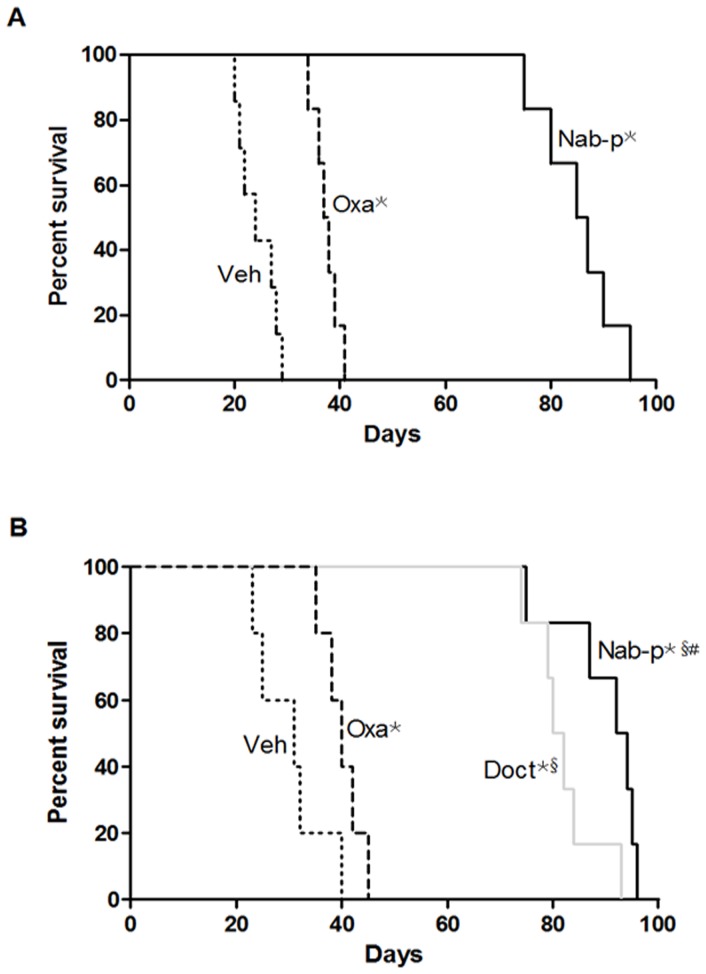
Effects of nab-paclitaxel therapy on the overall survival of mice. (A) Antitumor effect of nab-paclitaxel compared with oxaliplatin. SNU cells (40×10^6^) were injected intraperitoneally into SCID mice, followed by treatment after 2 weeks with nab-paclitaxel (10 mg/kg, 2 times a week) and oxaliplatin (5 mg/kg, 2 times a week) for 2 weeks. (B) Antitumor effect of nab-paclitaxel compared with oxaliplatin and docetaxel. SNU cells (40×10^6^) were injected intraperitoneally into SCID mice, followed by treatment after 2 weeks with nab-paclitaxel (10 mg/kg, 2 times a week), oxaliplatin (5 mg/kg, 2 times a week), or docetaxel (3 mg/kg, 2 times a week) for 2 weeks. The curve represents the animal survival time from the beginning of therapy. Symbol * represents significant difference compared with vehicle control (p<0.05), symbol ^§^ represents significant differences compared with oxaliplatin (p<0.05), and symbol ^#^ represents significant differences compared with docetaxel (p<0.05).

In the first survival study, the median survival of SCID-NOD mice was 24 days in the control group. This increased to 86 days after nab-paclitaxel treatment (p = 0.0004 versus control group, p = 0.0005 vs. oxaliplatin group) and to 37.5 days after oxaliplatin treatment (p = 0.0004 versus control group). The group survival range was also superior after nab-paclitaxel treatment (range: 75 to 95 days) compared to oxaliplatin treatment (range: 34 to 41 days) (Figure 6A).

In the second survival study, median survival of SCID-NOD mice was 31 days in the control group. This increased to 93 days after nab-paclitaxel treatment (p = 0.0007 vs. control and oxaliplatin group, and p = 0.0416 vs. docetaxel group), to 81 days after docetaxel treatment (p = 0.0007 vs. controls) and to 40 days by oxaliplatin treatment (p = 0.038 vs. control group). The group survival range was 75 to 96 days after nab-paclitaxel treatment compared to docetaxel treatment (range: 74 to 93 days) and oxaliplatin treatment (range: 35 to 45 days) (Figure 6B).

## Discussion

Advanced gastric cancer is life-threatening and represents a formidable treatment challenge. Traditional double or triple cytotoxic chemotherapy regimens have limited effects and can cause side effects and chemoresistance [42]–[44]. The present study clearly demonstrates that nab-paclitaxel has significantly stronger antitumor effects on gastric cancer cell lines than currently used cytotoxic agents oxaliplatin and epirubicin in vitro and in vivo, measured by antiproliferative effects, apoptosis, mitotic cell death, localized antitumor response and survival related to tumor burden.

In vitro study showed that nab-paclitaxel is highly potent in inhibiting cell proliferation for human gastric cell lines than oxaliplatin and epirubicin. SNU16 cells are more sensitive to nab-paclitaxel than NCI-N87 cells. Some studies found that stathmin was correlated with taxane resistance and stathmin knockdown can increase sensitivity to taxane [33], [36]. We tested the expression of stathmin and found no difference among these three cell lines. Interestingly, when we examined the expression of phospho-stathmin we found that the ability of nab-paclitaxel to inhibit in vitro cell proliferation was in the same rank order as the expression of phospho-stathmin in these three gastric cancer cells: SNU16> AGS > NCI-N87. It thus appeared initially that phospho-stathmin expression was correlated with nab-paclitaxel efficacy, lending some support to a hypothesis that phospho-stathmin can serve as a potential biomarker to predict nab-paclitaxel antitumor response in gastric cancer treatment.

We performed a NCI-N87 xenograft study to evaluate the antitumor effect of nab-paclitaxel. Rather unexpectedly, nab-paclitaxel significantly inhibited NCI-N87 xenograft tumor growth compared to control group. These somewhat discrepant results seem to suggest that the in vivo antitumor effect of nab-paclitaxel in gastric cancer can be independent from the baseline expression of phospho-stathmin or total stathmin. Since only three cell lines were tested, we cannot make a definite conclusion about the relationship between stathmin or phospho-stathmin and nab-paclitaxel efficacy in gastric cancer. Further research with stathmin knocked-outs should be useful to address this question and investigate the role of stathmin as biomarker for nab-paclitaxel efficacy.

In this study, we then compared antitumor effects of nab paclitaxel with oxaliplatin and epirubicin on SNU16 xenograft local tumors. Nab-paclitaxel showed stronger antitumor efficacy than oxaliplatin and epirubicin which was consistent with in vitro cell proliferation results and tumor tissue cell proliferation and apoptosis data. Although 5-fluouracil is the most established single-agent drug in chemotherapeutic gastric cancer care, the tumor response of 5-fluoruracil is reported to be inadequately predicted in SCID mouse models to the point where even no antitumor effects have been observed [45]. Cisplatin and oxaliplatin, more powerful inducers of apoptosis and also frequently used in gastric cancer chemotherapeutic treatment, have been chosen more often as agents for comparison purposes with new agents that are to be tested [22], [45]. Solvent-based paclitaxel has more side effects and did not add survival benefits compared to oxaliplatin in clinical treatment for gastric cancer [22]. In our studies, oxaliplatin did not differ statistically from epirubicin. We therefore choose oxaliplatin as a baseline agent in the animal survival study and found that nab-paclitaxel increased median mouse survival by about 40 days over oxaliplatin, a more than double effect than that achieved by oxaliplatin. Docetaxel appears to be the more active taxane, with more rapid cellular uptake and longer intracellular retention compared with solvent-based paclitaxel [46]. We performed another survival study to compare antitumor effects of nab-paclitaxel with docetaxel and found that the median survival time was slightly extended after nab-paclitaxel treatment (93 days vs. 81 days, p = 0.0416). Our results were consistent with results of a recent Phase II breast cancer trial which showed that 100 mg/m^2^ nab-paclitaxel had prolonged median overall survival (hazard ratio 0.575, p = 0.008) compared with docetaxel [47]. Based on our results, nab-paclitaxel showed stronger antitumor efficacy in experimental gastric cancer and appears to represent a reasonably potent new chemotherapeutic agent for clinical gastric cancer treatment.

Stathmin is an important microtubule microtubule-destabilizing phosphoprotein. When stathmin is phosphorylated, its microtubule-destabilizing activity decreased [32], [34] and this may improve sensitivity to taxane. Stathmin is phosphorylated mainly on the cyclin-dependent protein kinase (CDKs) binding site Ser38 in mitosis [32], so we focused our expression analyses on the ser38 residue. We found that nab-paclitaxel treatment increased phospho-stathmin expression in vitro and in vivo and was correlated with mitotic arrests, nuclear fragmentation, karyopyknosis and mitotic cell death. Mitotic cell death occurred in those cells with high expression of phospho-stathmin. Zhou et al. [33] found that down-regulated stathmin expression after STMN1 gene knockdown in hepatic carcinoma cell added 7.7-fold sensitivity to nab-paclitaxel and 2.7-fold sensitivity to solvent-based paclitaxel but no sensitivity to doxorubicin. The molecular nature that triggers cell death during prolonged mitotic arrest remains poorly defined [17], [32]. Spindle assembly checkpoints have long been thought to play a critical role during this process [48]. In this study, nab-paclitaxel induced phosphorylation of stathmin in gastric cancer cells, which is expected to stabilize the microtubule apparatus, which in turn can cause mitotic arrests and trigger cell death. Although these are early and preliminary data, some support for phospho-stathmin regulating nab-paclitaxel antitumor activity has been observed and deserves to be further evaluated.

In conclusion, the present study demonstrates that single-agent nab-paclitaxel had stronger antitumor activity in experimental gastric cancer than the current standard chemotherapeutic agents oxaliplatin and epirubicin, and appeared to be the superior taxane compared to docetaxel. This strong antitumor activity supports the rationale for clinical evaluation of nab-paclitaxel as promising microtubule-inhibitory agent in gastric cancer.

## Supporting Information

Figure S1 **Nab-paclitaxel leads to G2-M phase arrest in gastric cancer cells.** (A) Histograms show cell-cycle profiles of AGS and NCI-N87 cells at different times after nab-paclitaxel treatment. Gastric cancer cells were cultured for 24 hours and then treated with 100 nM nab-paclitaxel for 12 hours or 24 hours. Cell cycle analysis was performed by flow cytometry. Results shown are representative of two independent experiments. (B) Cell cycle distribution for gastric cancer cells treated by 100 nM nab-paclitaxel. Symbol * marks conditions under which cells had disintegrated.
(TIF)

Figure S2 **Cell cycle progression of gastric cancer cells treated by oxaliplatin, epirubicin and docetaxel.** (A) Histograms show cell-cycle profiles of SNU16 and AGS cells after oxaliplatin, epirubicin and docetaxel treatment. Gastric cancer cells were cultured for 24 hours and then treated with 10 μM nab-paclitaxel, oxaliplatin, epirubicin and docetaxel for 8 hours. Cell cycle was performed by flow cytometry. Results shown are representative of two independent experiments. (B) Cell cycle distribution for gastric cancer cells treated by 10 μM nab-paclitaxel, oxaliplatin, epirubicin and docetaxel.
(TIF)
