# Supplementary figures and images for: Superior Antitumor Activity of Nanoparticle Albumin-Bound Paclitaxel in Experimental Gastric Cancer
Source: PLoS One. 2013 Feb 27;8(2):e58037. doi: 10.1371/journal.pone.0058037 (PMC3584019; doi:10.1371/journal.pone.0058037)

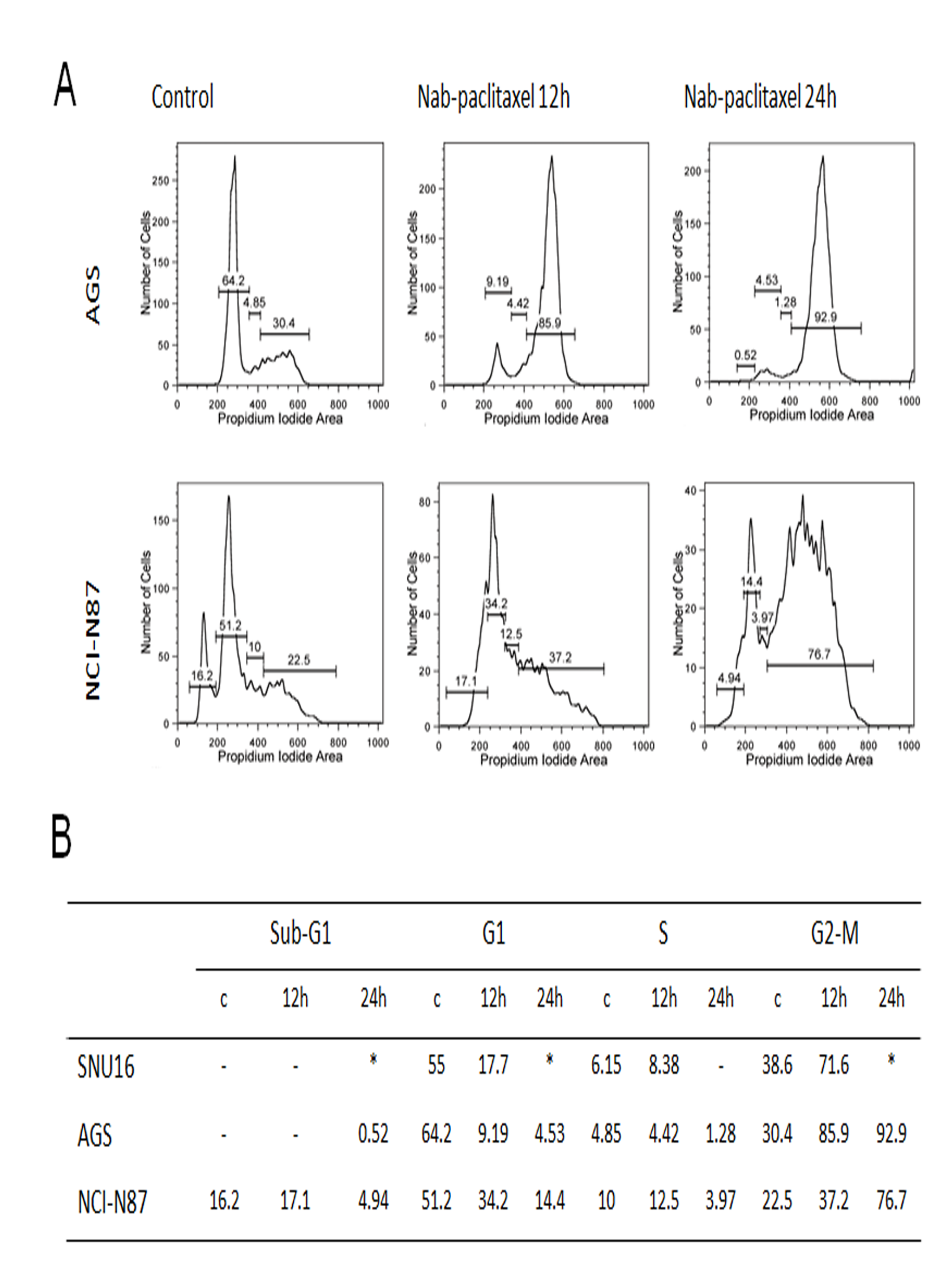

Supplement: Figure S1 — Nab-paclitaxel leads to G2-M phase arrest in gastric cancer cells. (A) Histograms show cell-cycle profiles of AGS and NCI-N87 cells at different times after nab-paclitaxel treatment. Gastric cancer cells were cultured for 24 hours and then treated with 100 nM nab-paclitaxel for 12 hours or 24 hours. Cell cycle analysis was performed by flow cytometry. Results shown are representative of two independent experiments. (B) Cell cycle distribution for gastric cancer cells treated by 100 nM nab-paclitaxel. Symbol * marks conditions under which cells had disintegrated. (TIF) [file pone.0058037.s001.tif]

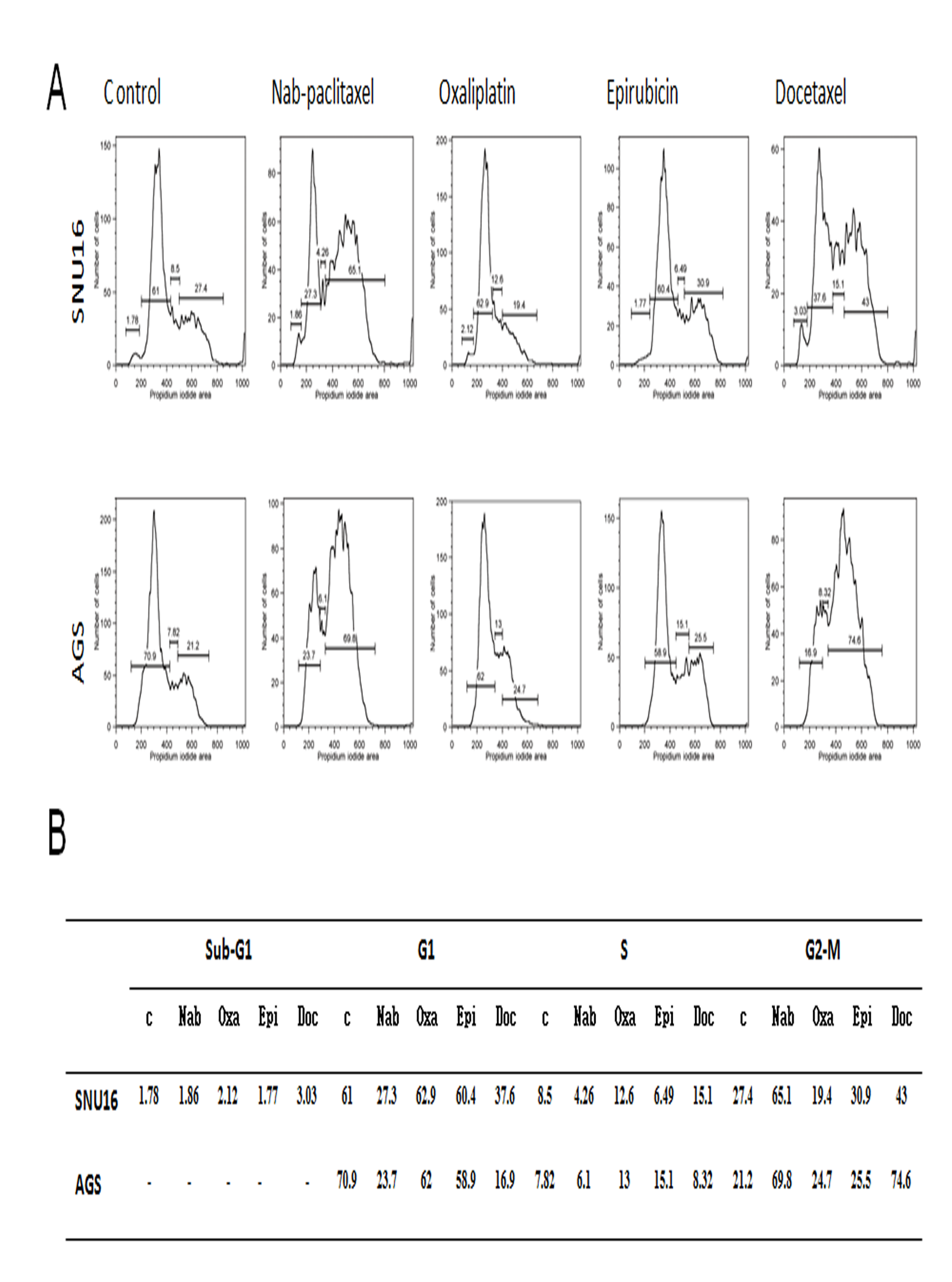

Supplement: Figure S2 — Cell cycle progression of gastric cancer cells treated by oxaliplatin, epirubicin and docetaxel. (A) Histograms show cell-cycle profiles of SNU16 and AGS cells after oxaliplatin, epirubicin and docetaxel treatment. Gastric cancer cells were cultured for 24 hours and then treated with 10 μM nab-paclitaxel, oxaliplatin, epirubicin and docetaxel for 8 hours. Cell cycle was performed by flow cytometry. Results shown are representative of two independent experiments. (B) Cell cycle distribution for gastric cancer cells treated by 10 μM nab-paclitaxel, oxaliplatin, epirubicin and docetaxel. (TIF) [file pone.0058037.s002.tif]
